# Standardized Interpretation of Chest Radiographs in Cases of Pediatric Pneumonia From the PERCH Study

**DOI:** 10.1093/cid/cix082

**Published:** 2017-05-27

**Authors:** Nicholas Fancourt, Maria Deloria Knoll, Breanna Barger-Kamate, John de Campo, Margaret de Campo, Mahamadou Diallo, Bernard E. Ebruke, Daniel R. Feikin, Fergus Gleeson, Wenfeng Gong, Laura L. Hammitt, Rasa Izadnegahdar, Anchalee Kruatrachue, Shabir A. Madhi, Veronica Manduku, Fariha Bushra Matin, Nasreen Mahomed, David P. Moore, Musaku Mwenechanya, Kamrun Nahar, Claire Oluwalana, Micah Silaba Ominde, Christine Prosperi, Joyce Sande, Piyarat Suntarattiwong, Katherine L. O’Brien

**Affiliations:** 1 Department of International Health, International Vaccine Access Center, Johns Hopkins Bloomberg School of Public Health, Baltimore, Maryland;; 2 Murdoch Childrens Research Institute, and; 3 Royal Children’s Hospital, Melbourne, Australia;; 4 Department of Pediatrics, Division of Emergency Medicine, Johns Hopkins School of Medicine, Baltimore, Maryland;; 5 Spokane Emergency Physicians, Washington;; 6 Department of Radiology, Melbourne University, Australia;; 7 Centre pour le Développement des Vaccins (CVD-Mali), Bamako;; 8 Medical Research Council Unit, Basse, The Gambia;; 9 Division of Viral Diseases, National Center for Immunization and Respiratory Diseases, Centers for Disease Control and Prevention, Atlanta, Georgia;; 10 Oxford University Hospitals NHS Trust, United Kingdom;; 11 Kenya Medical Research Institute–Wellcome Trust Research Programme, Kilifi;; 12 Center for Global Health and Development, Boston University School of Public Health, Massachusetts;; 13 Queen Sirikit National Institute of Child Health, Bangkok, Thailand;; 14 Medical Research Council, Respiratory and Meningeal Pathogens Research Unit, and; 15 Department of Science and Technology/National Research Foundation, Vaccine Preventable Diseases Unit, University of the Witwatersrand, Johannesburg, South Africa;; 16 International Centre for Diarrhoeal Disease Research, Bangladesh (icddr,b), Dhaka and Matlab;; 17 Department of Diagnostic Radiology, and; 18 Department of Paediatrics and Child Health, Chris Hani Baragwanath Academic Hospital and University of the Witwatersrand, Johannesburg, South Africa;; 19 Department of Pediatrics, University Teaching Hospital, Lusaka, Zambia; and; 20 Aga Khan University Hospital, Nairobi, Kenya

**Keywords:** observer variation, chest radiograph, pneumonia, pediatrics, diagnosis.

## Abstract

**Background.:**

Chest radiographs (CXRs) are a valuable diagnostic tool in epidemiologic studies of pneumonia. The World Health Organization (WHO) methodology for the interpretation of pediatric CXRs has not been evaluated beyond its intended application as an endpoint measure for bacterial vaccine trials.

**Methods.:**

The Pneumonia Etiology Research for Child Health (PERCH) study enrolled children aged 1–59 months hospitalized with WHO-defined severe and very severe pneumonia from 7 low- and middle-income countries. An interpretation process categorized each CXR into 1 of 5 conclusions: consolidation, other infiltrate, both consolidation and other infiltrate, normal, or uninterpretable. Two members of a 14-person reading panel, who had undertaken training and standardization in CXR interpretation, interpreted each CXR. Two members of an arbitration panel provided additional independent reviews of CXRs with discordant interpretations at the primary reading, blinded to previous reports. Further discordance was resolved with consensus discussion.

**Results.:**

A total of 4172 CXRs were obtained from 4232 cases. Observed agreement for detecting consolidation (with or without other infiltrate) between primary readers was 78% (κ = 0.50) and between arbitrators was 84% (κ = 0.61); agreement for primary readers and arbitrators across 5 conclusion categories was 43.5% (κ = 0.25) and 48.5% (κ = 0.32), respectively. Disagreement was most frequent between conclusions of other infiltrate and normal for both the reading panel and the arbitration panel (32% and 30% of discordant CXRs, respectively).

**Conclusions.:**

Agreement was similar to that of previous evaluations using the WHO methodology for detecting consolidation, but poor for other infiltrates despite attempts at a rigorous standardization process.

The chest radiograph (CXR) is a valuable diagnostic tool for pneumonia, both as part of clinical management [1] and for determining case status in epidemiological studies [2]. CXRs can be archived and systematically evaluated, enabling cross-study comparisons. However, CXR interpretations are subjective, making it difficult to achieve measurements that are reproducible, reliable, and valid [3–5]. Acknowledging this, the World Health Organization (WHO) developed a standardized methodology for the interpretation of pediatric CXRs (the “WHO methodology”), designed to optimize the identification of *Streptococcus pneumoniae* and *Haemophilus influenzae* type b (Hib) pneumonia in vaccine trials [2, 6]. The WHO methodology has since been adopted by many studies of vaccine efficacy and effectiveness [7–11], a trial of indoor air pollution reduction [12], incidence and surveillance studies [13–15], and descriptive epidemiology of pneumonia cases [16, 17]. Despite widespread use, there has been no evaluation of how best to implement the WHO methodology, especially beyond its initial application in vaccine trials.

Here we describe the process for CXR interpretation in a large childhood pneumonia study, evaluate the standardization of readers and observer variability, and assess the process of arbitration for discordant interpretations.

## METHODS

### Data Collection

Pneumonia Etiology Research for Child Health (PERCH) is a multicountry, standardized, case-control study of the causes and risk factors of childhood pneumonia [18]. A total of 4232 cases of hospitalized, WHO-defined severe or very severe pneumonia in children aged 1–59 months were enrolled from August 2011 to January 2014. Nine sites in 7 countries were chosen to be representative of the epidemiological contexts where pneumonia is most prevalent: Dhaka and Matlab, Bangladesh; Basse, The Gambia; Kilifi, Kenya; Bamako, Mali; Soweto, South Africa; Nakhon Phanom and Sa Kaeo, Thailand; and Lusaka, Zambia. The institutional review board or ethical review committee approved the study protocol at each of the 7 institutions and at the Johns Hopkins Bloomberg School of Public Health. Parents or guardians of participants provided written informed consent.

A CXR was sought from each case as soon as practical after clinical evaluation and study enrollment; some children had repeat CXRs if clinically indicated. In cases where a CXR was not obtained, the reason was recorded. All CXRs were taken in either anterior-posterior or posterior-anterior format as required by the WHO methodology [2]. Most sites used digital CXR imaging equipment, except Zambia and Matlab where analog techniques were used. The Gambian site used an analog machine when there were technical problems with their digital system. At Nakhon Phanom and South Africa, analog CXRs were performed for 11 and 8 months, respectively, before digital systems were installed. All analog images were scanned into digital format [19]. All sites were assessed as meeting quality and safety requirements prior to study enrollment.

### Chest Radiograph Interpretation

Two members from each of the 7 study sites (5 radiologists and 9 pediatricians with 0–28 years postspecialization experience) formed the CXR reading panel. Four additional radiologists (3 with extensive WHO methodology experience) from Australia, Kenya, and the United Kingdom formed an arbitration panel to interpret CXRs discordant at the initial interpretation, and ensured consistency to previous studies by using a common arbitration process [2]. Members of the arbitration panel also provided a 2-day, in-person training workshop for the reading panel. To ensure this training was optimized for PERCH, the arbitration panel met first to calibrate the application of the WHO definitions to PERCH CXRs. Three members of the reading panel who were unable to attend the training viewed recorded lectures and met with another member of the reading panel to review key concepts. Prior to interpreting PERCH study CXRs, all readers were assessed by interpreting 20 randomly selected WHO reference CXRs. Readers were required to correctly identify the reference conclusion for ≥50% of all images, ≥66% of images with consolidation, and ≥66% of normal images. Repeat training and assessment with additional sets of 20 images was performed until standardization was achieved. Continuing education was provided through monthly emails that reviewed key teaching points, and a voluntary reassessment with the first set of 20 WHO images.

**Figure 1.**
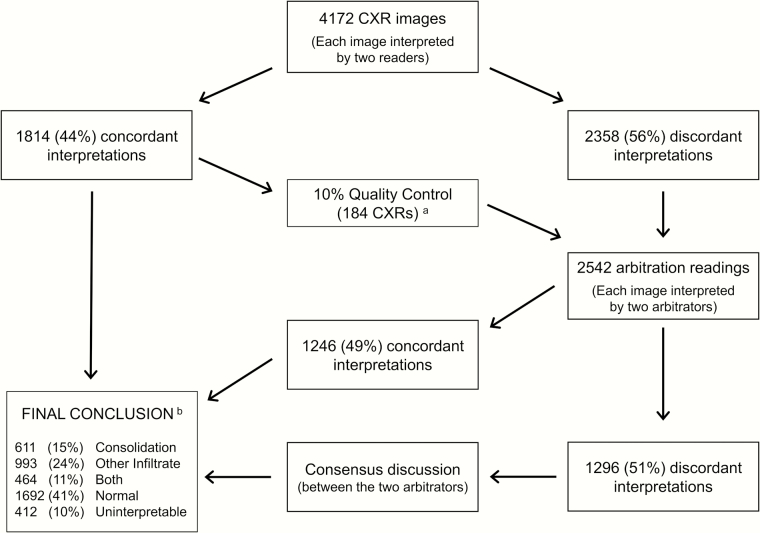
Interpretation process for chest radiographs (CXRs) in the Pneumonia Etiology Research for Child Health (PERCH) project. ^a^Arbitration results for quality control images were not used to determine the final conclusion. ^b^Final conclusion represents the conclusion reached for each of the 4172 CXRs and not the distribution of CXR diagnoses for the 4232 enrolled cases as some cases have multiple CXRs interpreted and some missing.

Figure 1 shows the process for interpretation of CXRs. Arbitrators were blinded to previous conclusions except at final consensus discussions. Table 1 shows the classification of findings, conclusions derived from these findings, and the arbitration process used [6]. The WHO methodology was optimized for “any consolidation” (also termed “primary endpoint pneumonia” as a specific reference to the outcome of interest in vaccine trials) and thus this conclusion is frequently evaluated. Also outlined in Table 1 are alternate conclusions and arbitration processes used to evaluate the effects of 4 different interpretation methods on observer agreement, the distribution of conclusions, and the number of interpretations required.

**Table 1.** Classification of Findings and Conclusions for the Standardized Interpretation of Chest Radiographs

Classification			Definition				
Findings (adapted from Cherian et al ^[6]^)	Consolidation^a^		A dense or fluffy opacity that occupies a portion or whole of a lobe or of the entire lung that may or may not contain air bronchograms^b^				
	Other infiltrate		Linear and patchy densities (interstitial infiltrate) in a lacy pattern involving both lungs, featuring peribronchial thickening and multiple areas of atelectasis; it also includes minor patchy infiltrates that are not of sufficient magnitude to constitute primary endpoint consolidation, and small areas of atelectasis which in children may be difficult to distinguish from consolidation				
	Pleural effusion		Presence of fluid in the lateral pleural space between the lung and chest wall; in most cases, this will be seen at the costophrenic angle or as a layer of fluid adjacent to the lateral chest wall; this does not include fluid seen in the horizontal or oblique fissures				
	Uninterpretable		Features of the image are not interpretable with respect to presence or absence of consolidation and/or other infiltrate^c^				
			Interpretation process				
			PERCH^d^	Single arbitrator	4 conclusions	Vaccine trial	Any abnormality
Conclusions (based on the above findings)	Only consolidation or pleural effusion without other infiltrate		X	X			
	Any consolidation or pleural effusion with or without other infiltrate				X	X	
	Other infiltrate without consolidation		X	X	X	X	
	Both consolidation and other infiltrate		X	X			
	Any consolidation or other infiltrate						X
	Normal (no consolidation, other infiltrate, pleural effusion, or uninterpretable findings)		X	X	X	X	X
	Uninterpretable for consolidation and/or other infiltrate		X	X	X		X
	Uninterpretable for consolidation only^e^					X	
Arbitration	Arbitration panel or single arbitrator^f^		Panel	Single	Panel	Panel	Panel

Abbreviation: PERCH, Pneumonia Etiology Research for Child Health.

^a^ The presence of consolidation or pleural effusion was described in the World Health Organization methodology as “primary endpoint pneumonia” rather than “consolidation” as a specific reference to the outcome of interest in bacterial vaccine trials. The descriptive term “consolidation” is preferred in a more general epidemiologic context such as PERCH.

^b^Atelectasis of an entire lobe that produces an opacity and a positive silhouette sign with the mediastinal border was considered to be consolidation.

^c^Where any reader or arbitrator reported a finding of consolidation alongside a finding of uninterpretable for other infiltrate (or vice versa) the interpretation was consolidation (or other infiltrate). That is, where a pathological finding was reported this was prioritized over an uninterpretable finding when determining the interpretation for the image.

^d^This interpretation process was used to define chest radiograph (CXR) outcomes for PERCH cases. Other processes are examined here to illustrate effects of different interpretation methods on CXR outcomes.

^e^For 64 images where the altered definition of uninterpretable produced discordant interpretations by 2 readers or 2 arbitrators, and no further arbitration interpretations were available, conclusions were imputed based on the distribution of conclusions from arbitration of uninterpretable images using the PERCH definitions.

^f^“Arbitration panel” = where the primary reading resulted in discordant interpretations for any conclusions, the CXR was randomized and independently interpreted by 2 arbitrators. Where these arbitrators’ conclusions were discordant, the 2 arbitrators reached agreement through a consensus discussion. Arbitrators were aware of previous conclusions at the final arbitration discussion only; “Single arbitrator” = where the primary reading resulted in discordant interpretations for any conclusions, an arbitration decision was sought from a single interpretation by the most experienced arbitrator, or by the next most experienced arbitrator when available, or by the third most experienced arbitrator for remaining images. Arbitrators were not aware of previous conclusions.

### Analysis

We assessed agreement for the primary reading and arbitration panels, as well as separately for each member of the primary reading panel. Observer agreement was evaluated by observed percentage agreement and the kappa statistic (κ), which provides a measure of agreement adjusted for chance agreement [20]. Fleiss’ κ was used for interobserver calculations because PERCH used randomized reader-pairs rather than observers with a constant identity across interpretations [20]. Cohen’s κ was used for intraobserver assessment of repeat standardization assessments, and for interobserver calculations for individual conclusions to allow calculation of confidence intervals. For analyses of individual conclusions, a κ adjusted for prevalence and differences in each reader’s distribution of findings (also known as marginal distributions) was also calculated [21, 22]. Because uninterpretable images are assumed to be a consequence of the imaging process and image quality may contribute to variability in interpretation, for some analyses images with one or more interpretations of uninterpretable are excluded, as is common in evaluating observer agreement for CXRs [3, 6]. The χ^2^ goodness-of-fit test was used to assess the distribution of final arbitration discussion conclusions that agreed with each arbitrator’s initial interpretation, using equal proportions (25%) as expected values.

Data exploration and analyses were completed using Stata software version 12.1 (StataCorp, College Station, Texas).

## RESULTS

Seven (50%) readers passed the standardization assessment on the first attempt, 3 on a second attempt, and 4 on a third attempt. The voluntary standardization assessment 8 months after interpretations began was completed by 11 of 14 readers, with intraobserver agreement for the identification of any consolidation in the WHO reference CXRs ranging from 85% to 100% (mean, 91%) and κ values from 0.63 to 1.0 (mean, 0.82).

Of 4232 PERCH cases, 4011 (95%) provided 4172 CXRs, with 120 cases providing >1 image. Of the 221 cases without a CXR, 92 (42%) were because the child died before a CXR could be taken, 23 (10%) had been discharged, 36 (16%) encountered equipment or operator errors, and 70 (32%) were for unknown reasons.

Observed agreement from the interpretation process is summarized in Figure 1. Of the 4172 CXRs reviewed there was at least one interpretation of uninterpretable for 675 (16%) of primary readings and 497 (21%) of arbitration readings. Among images without an uninterpretable reading (ie, “interpretable” CXRs), interobserver agreement was highest for the detection of any consolidation for both the primary reading panel (78% observed agreement; κ = 0.50; 95% confidence interval [CI], .47–.53) and arbitration panel (84%; κ = 0.61; 95% CI, .56–.65; Table 2). The adjusted κ for any consolidation was 0.56 and 0.67 for the primary and arbitration panels, respectively. There was variation in observer agreement for the detection of any consolidation between sites; however, much of this variation was not present after κ values were adjusted for prevalence and marginal distributions (Supplementary Figure 2). Differences between observed agreement and κ values were influenced by the prevalence of each conclusion more than the different marginal distributions between readers (Supplementary Table 3). Considering agreement across all 5 conclusion categories, 1814 CXRs had a concordant interpretation by the primary reading panel (44% observed agreement; κ = 0.25; 95% CI, .23–.27). Of 2358 CXRs interpreted by arbitrators (excluding quality control images), 1144 had a concordant interpretation (49%; κ = 0.32; 95% CI, .30–.34). Among 2358 CXRs reviewed at arbitration, there was agreement with one of the primary readers’ interpretations by one or both arbitrators for 1114 (47%) and 930 (40%) CXRs, respectively.

**Table 2.** Observer Agreement for Individual Conclusions (Present or Absent) for the 14-Member Primary Reading Panel and the 4-Member Arbitration Panel, Excluding Images for Which Either Reader/Arbitrator Interpreted as Uninterpretable

	Observer Agreement							
	Primary Readings (n = 3497)				Arbitration Readings (n = 1861)^a^			
Conclusion	Observed Agreement (%)	κ	(95% CI)	Adjusted κ^b^	Observed Agreement (%)	κ	(95% CI)	Adjusted κ^b^
Only consolidation	80.0	0.33	(.30–.37)	0.60	82.5	0.32	(.28–.37)	0.65
Other infiltrate	66.5	0.15	(.12–.18)	0.33	67.8	0.25	(.20–.29)	0.36
Both	80.3	0.21	(.18–.24)	0.61	82.6	0.30	(.25–.34)	0.65
Normal	68.9	0.35	(.32–.38)	0.38	77.1	0.52	(.47–.57)	0.54
Any consolidation^c^	77.8	0.50	(.47–.53)	0.56	83.6	0.61	(.56–.65)	0.67
Left	94.8	0.39	(.36–.42)	0.90	92.7	0.42	(.38–.47)	0.85
Right	82.6	0.46	(.43–.50)	0.65	88.6	0.52	(.48–.57)	0.77
Bilateral	91.6	0.37	(.34–.40)	0.84	92.2	0.49	(.44–.53)	0.84
Case age, mo^c^								
1–5	76.7	0.49	(.44–.54)	0.53	83.9	0.64	(.57–.72)	0.68
6–11	78.1	0.52	(.45–.59)	0.56	83.3	0.59	(.50–.69)	0.67
≥12	78.8	0.49	(.44–.54)	0.58	83.5	0.57	(.49–.64)	0.67
Equipment and processing technique^c^								
Digital	78.5	0.51	(.47–.55)	0.57	84.8	0.62	(.56–.67)	0.70
Analog	76.0	0.48	(.41–.54)	0.52	80.3	0.58	(.49–.66)	0.61
Time since standardization training^c,d^								
≤10 mo	81.5	0.59	(.54–.64)	0.63	83.0	0.61	(.52–.70)	0.66
>10 mo	74.9	0.43	(.38–.47)	0.50	83.8	0.61	(.55–.66)	0.68
Readers’ specialty and years of postspecialization experience^c^								
Pediatrics	76.4	0.50	(.45–.55)	0.53	…	…	…	…
Radiology	83.5	0.53	(.44–.63)	0.67	…	…	…	…
≤5 y	76.6	0.48	(.41–.55)	0.53	…	…	…	…
>5 y	80.0	0.52	(.45–.59)	0.60	…	…	…	…

Abbreviation: CI, confidence interval.

^a^Excludes 184 quality control images interpreted by arbitrators.

^b^Adjusted for prevalence and bias [22].

^c^Stratified results are presented for “any consolidation” because this is the primary endpoint of interested under most applications of the World Health Organization methodology.

^d^Total time for interpretations by the primary reading panel was 20 months. The arbitration panel did not undergo assessments for standardization but did participate and lead the training process.

**Figure 2.**
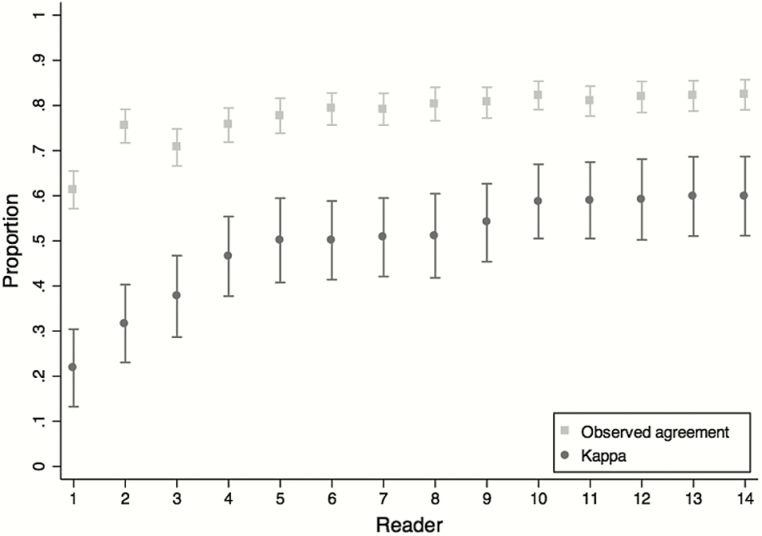
Observer agreement for individual readers for the finding of any consolidation, excluding images for which either reader concluded as uninterpretable (range, 440–568).

Each of the 14 readers interpreted an average of 598 images (range, 535–659). Agreement for individual readers was highest for any consolidation (observed agreement 61%–81%; κ = 0.22–0.60; Figure 2). Considering all 5 conclusions, observed agreement for individual readers ranged from 30% to 55% (κ = 0.07–0.32). The reader with the lowest agreement across these 5 conclusions was an outlier, with results (27%; κ = 0.07; 95% CI, .02–.11) significantly lower than the reader with the next lowest κ (45%; κ = 0.19; 95% CI, .14–.23). These 2 readers with the lowest κ values had no prior experience in the WHO methodology and did not attend the in-person training. The most frequent type of discordance was between normal and other infiltrate, which accounted for 743 of 2358 (32%) CXRs discordant at the primary interpretation and 360 of 1214 (30%) CXRs discordant at arbitration (Table 3).

**Table 3.** Summary of Discordant and Concordant Conclusions for Either the 2 Randomly Assigned Readers or the 2 Randomly Assigned Arbitrators

	Discordant Conclusions^a^, No. (Row %)										
	Normal/ Infiltrate	Infiltrate/ Consol.	Normal/ Consol.	Consol./ Uninterp.	Both/ Consol.	Both/ Infiltrate	Both/ Normal	Both/ Uninterp.	Normal/ Uninterp.	Infiltrate/ Uninterp.	Total^b^
Readers	743 (31.5)	198 (8.4)	196 (8.3)	126 (5.3)	306 (13.0)	232 (9.8)	149 (6.3)	47 (2.0)	264 (11.2)	97 (4.1)	2358
Arbitrators	360 (29.7)	108 (8.9)	46 (3.8)	62 (5.1)	171 (14.1)	130 (10.7)	21 (1.7)	23 (1.9)	181 (14.9)	112 (9.2)	1214
	Concordant Conclusions, No. (Row %)										
	Consol.	Infiltrate	Both	Normal	Uninterp.	Total^b^					
Readers	294 (16.2)	361 (19.9)	164 (9.0)	854 (47.1)	141 (7.8)	1814					
Arbitrators	121 (10.6)	276 (24.1)	107 (9.4)	521 (45.5)	119 (10.4)	1144					

^a^Both, consolidation and other infiltrate; Consol., consolidation; Infiltrate, other infiltrate; Uninterp., uninterpretable.

^b^Among arbitrators, excludes 184 quality control images.

The 4 arbitrators interpreted an average of 1179 images (range, 1175–1187). Of these interpretations, 561–647 (range, 48%–55%) were discordant with the other arbitrator. We expected that each arbitrator would have an equal proportion (25%) of all consensus discussion conclusions agree with their initial interpretation; however, one arbitrator had a lower proportion (15%) and one a higher proportion (34%, *P* < .0001). The final arbitration discussion conclusion was different from both arbitrators’ initial interpretations for 89 of 1214 (7%) CXRs.

The arbitration panel reviewed 184 CXRs for quality control. Of the 44 CXRs concordant for any consolidation at the primary reading panel, there was agreement on this conclusion by both arbitrators for 30 (68%) CXRs and agreement by at least one arbitrator for 41 (93%; Table 4). Across all 5 conclusions, there was concordance between arbitrators for 102 (55%) images and, of these, 83 (81%) had the same conclusion as the primary reading panel. After final arbitration discussions, there was concordance between the conclusion of the readers and arbitrators in 129 of the 184 CXRs (70%).

**Table 4.** Results of the Quality Control Process (Arbitration of a Random Selection of 10% Concordant Images for Each Conclusion from the Primary Reading)

	Arbitrators’ Conclusions, No. (Row %)						
Readers’ Conclusion	Both Agree With Readers		One Agrees & One Disagrees With Readers		Both Disagree With Readers		Total
Only consolidation							
* *Yes	11	(37.9)	12	(41.4)	6	(20.7)	29
* *No	2	(1.3)	15	(9.7)	138	(89.0)	155
* *Total	13		27		144		184
Other infiltrate							
* *Yes	9	(25.7)	17	(48.6)	9	(25.7)	35
* *No	3	(2.0)	26	(17.4)	120	(80.5)	149
* *Total	12		43		129		184
Both							
* *Yes	2	(13.3)	9	(60.0)	4	(26.7)	15
* *No	4	(2.4)	12	(7.1)	153	(90.5)	169
* *Total	6		21		157		184
Normal							
* *Yes	52	(60.5)	26	(30.2)	8	(9.3)	86
* *No	8	(8.2)	20	(20.4)	70	(71.4)	98
* *Total	60		46		78		184
Uninterpretable							
* *Yes	9	(47.4)	5	(26.3)	5	(26.3)	19
* *No	2	(1.2)	22	(13.3)	141	(85.5)	165
* *Total	11		27		146		184
Any consolidation							
* *Yes	30	(68.2)	11	(25.0)	3	(6.8)	44
* *No	4	(2.9)	7	(5.0)	129	(92.1)	140
* *Total	34		18		132		184

We evaluated 4 different interpretation processes that had alternate conclusions or arbitration methods and assessed their effect on the distribution of conclusions, observer agreement, and total number of interpretations compared to the PERCH interpretation process (Table 5). The process used in the vaccine trials [23], which considered images discordant between other infiltrate/normal or other infiltrate/uninterpretable as other infiltrate (ie, no arbitration), resulted in 23% of images with final conclusions different from those obtained under the PERCH process. As expected, this process also identified the highest proportion of images with other infiltrate (39% vs 24% using the PERCH process). Although a process using a single arbitration interpretation produced a similar distribution of conclusions to the PERCH process, 16% of CXRs had a different conclusion. Using a majority decision from the primary reading interpretations and that of a single arbitrator left 480 of 4172 (12%) CXRs without a conclusion under the PERCH process and 226 (5%) without a conclusion under the vaccine trial process (data not shown). The various processes also required different total numbers of interpretations; the PERCH process required the most, while the single arbitration process and the vaccine trial process required 25% and 24% fewer interpretations, respectively.

**Table 5.** Comparison of Pneumonia Etiology Research for Child Health (PERCH) Study and Alternate Processes for Chest Radiographic Interpretation (Includes Multiple Images on 120 Cases)

	Conclusions^a^, No. (%)								Agreement, % (κ)		Total
Interpretation Process	Only Consol.	Other Infiltrate	Both	Uninterp.	Normal	Any Consol.	Abnormal	Conclusion Changed	Readers	Arbitrators	No. of Readings (% Difference)
All 4172 CXRs											
PERCH^b^	611 (14.7)	993 (23.8)	464 (11.1)	412 (9.9)	1692 (40.6)	1075 (25.8)	2068 (49.6)	Ref	43.5 (0.25)	48.5 (0.32) n = 2358	14274 (Ref)
Single arbitration^c^	638 (15.3)	979 (23.5)	414 (9.9)	488 (11.7)	1653 (39.6)	1052 (25.2)	2028 (48.6)	680 (16.3)	43.5 (0.25)	…	10702 (25.0)
4 conclusions^d^	…	950 (22.8)	…	398 (9.5)	1684 (40.4)	1140 (27.3)	2090 (50.1)	65 (1.6)	50.8 (0.31)	52.8 (0.33) n = 2052	13417 (6.0)
Vaccine trial^e^	…	1615 (38.7)	…	155 (3.7)	1330 (31.9)	1072 (25.7)	2687 (64.4)	938 (22.5)	53.2 (0.32)	52.2 (0.34) n = 1113	10866 (23.9)
Any abnormality^f^	…	…	…	376 (9.0)	1612 (38.6)	…	2184 (52.4)	116 (2.8)	61.1 (0.32)	60.0 (0.35) n = 1622	12237 (14.3)

Abbreviations: CXR, chest radiograph; PERCH, Pneumonia Etiology Research for Child Health.

^a^Both, consolidation and other infiltrate; Consol., Consolidation; Uninterp., Uninterpretable. “Any consolidation” combines images concluded as “only consolidation” or “both consolidation and other infiltrate”; “Abnormal” combines images concluded as only consolidation,” “other infiltrate,” or “both”; “Conclusion changed” compares to PERCH process results, reclassified to the conclusion categories of the comparison process where necessary.

^b^Five conclusions (consolidation only, other infiltrate only, both consolidation and other infiltrate, normal, uninterpretable for consolidation and/or other infiltrate); 2 arbitrators; final arbitration discussion.

^c^Five conclusions (consolidation only, other infiltrate only, both consolidation and other infiltrate, normal, uninterpretable for consolidation and other infiltrate); single arbitration from the most experienced arbitrator when available, or from the next most experienced arbitrator when available, or from the third most experienced arbitrator for remaining images.

^d^Four conclusions (any consolidation, other infiltrate only, normal, uninterpretable for consolidation and/or other infiltrate); 2 arbitrators; final arbitration discussion.

^e^Four conclusions (any consolidation, other infiltrate only, normal, uninterpretable for consolidation only); disagreement between other infiltrate and normal, or other infiltrate and uninterpretable, is concluded as positive for other infiltrate; 2 arbitrators; final arbitration discussion. For 64 images where the altered definition of uninterpretable produced discordant interpretations by 2 readers or 2 arbitrators, and no further arbitration interpretations were available, conclusions were imputed based on the distribution of conclusions from arbitration of uninterpretable images using the PERCH definitions.

^f^Three conclusions (any consolidation and/or other infiltrate, normal, uninterpretable for consolidation and/or other infiltrate); 2 arbitrators; final arbitration discussion.

## DISCUSSION

This study is the largest published evaluation of the WHO methodology, and one of few studies where standardization has been attempted across multiple sites with different epidemiological characteristics. Achieving standardization is important to provide confidence in the use of CXR results, including the interpretation of pneumonia etiology. Our results show measures of observer agreement for the detection of any consolidation that are consistent with other high-quality studies of childhood pneumonia [9, 13, 24], and similar to other subjective diagnostic tests, such as cervical cytopathology [25] and prostatic histopathology [26]. Our experience reaffirms findings that observer agreement is best for consolidation and poorest for findings of other infiltrate [3, 27]. The interpretation of observer variability requires consideration of study-specific factors that can influence κ, such as the prevalence of the conclusion under evaluation. Detailed understanding of the core components of the CXR interpretation process informs wider PERCH analyses and the transition of the WHO methodology from vaccine trials to other epidemiological contexts.

Standardized interpretation of CXRs is important to ensure that differences between sites or across time are not due to differences in CXR interpretation but to differences in the case mix of enrolled children. We minimized bias by ensuring readers did not interpret CXRs from their own site. This is important for a multisite study like PERCH, as comparisons by site will be central to some analyses. Our structured training process aimed to achieve a common standard of interpretation with the WHO methodology, calibrated to CXRs from the PERCH study. Although readers did not have to correctly interpret 100% of test images to be eligible to interpret PERCH CXRs, the requirements were pragmatic but robust, with several readers requiring repeated attempts to pass. However, our ability to evaluate whether the training itself actually improved individual ability was limited because there were no pretraining assessments and the number of images interpreted for assessments was small. Observer agreement for the primary reading panel declined between the first and second halves of the interpretation process, suggesting the readers had increasing difficulty in applying the interpretation criteria. Future studies may benefit from continuing education and regular standardization assessments.

The WHO methodology was designed to optimize the detection of any consolidation (termed primary endpoint pneumonia for the vaccine trials), and this conclusion had the highest level of agreement in our study, similar to other pneumonia studies [9, 13, 23, 24] and evaluations of the WHO methodology [3, 27]. Interobserver agreement for any consolidation in both the Californian and Gambian pneumococcal conjugate vaccine (PCV) trials was κ = 0.58 (data was not reported for other trials) [9, 23]. Similarly, a Mozambique pneumonia incidence study had an agreement of 77% (κ = 0.52) for any consolidation [13]. In an antibiotic treatment study in Brazil, agreement for the detection of any consolidation or other infiltrate was 87% (κ = 0.68). This higher κ likely reflects a case mix with a higher prevalence of consolidation because of an enrollment criterion requiring the presence of CXR infiltrates [24].

Relying solely on consolidation may underestimate burden of disease [28], as suggested by estimates from the South African PCV trial where only 38% of children with pneumococcal pneumonia were thought to have CXR consolidation [29]. While study methods and case selection criteria can influence prevalence estimates of consolidation [23, 30], PERCH used a rigorously standardized study protocol and demonstrated a varied prevalence between sites [31]. Other radiographic appearances also capture cases of true pneumonia, pneumococcal or otherwise. Unfortunately, agreement on the presence of other infiltrates is more difficult to achieve [3, 27]; our results show lowest agreement for a finding of other infiltrate (Table 2) and that discordance is most common between interpretations of normal and other infiltrate (Table 3). The limitation of the WHO methodology in identifying nonconsolidation findings is particularly important in contexts where the prevalence of consolidation is low and milder radiographic changes predominate, such as areas with access to early antibiotic therapy or widespread use of pneumococcal and Hib conjugate vaccines.

Despite some consistency between studies in observer agreement for any consolidation, it can be misleading to compare κ values without reference to differences in the prevalence of the conclusion under evaluation [22]. This can arise when comparing results for different CXR definition categories or between epidemiological contexts. We observed the paradox of prevalence unexpectedly altering κ values for any consolidation and only consolidation where approximately 80% agreement was observed for both but κ was 0.50 and 0.33, respectively, owing to the prevalence of any consolidation being closer to 50% (Table 2 and Supplementary Table 3). A paradoxically high κ can also be produced if the readers conclude a different proportion of positive findings, although we did not observe this (Supplementary Table 3). Nonetheless, examining differences in marginal proportions offers an important check to demonstrate the interchangeability of readers [22], particularly for a large panel of readers from different regions with a range of professional experience. Agreement will also decrease as the number of conclusion categories increases, explaining why agreement was higher for the any abnormality interpretation process (which had 3 conclusion categories) than the PERCH interpretation process (which had 5 conclusion categories; Table 4). Despite this, our results show consistency in the proportion of any consolidation (range, 25%–27%) identified by 4 different interpretation processes (Table 4).

Determining the “best” method for arbitration depends on the desire to maximize accuracy of interpretation of pneumonia cases within the study, the ability to standardize methods across studies to facilitate between-study comparisons, and financial and logistical constraints. Use of a separate, common, arbitration panel was established in the WHO methodology [2] and adopted for vaccine trials [7, 8, 11, 32] to ensure consistency between studies. Using arbitrators with extensive experience in the WHO methodology is favored over a consensus discussion between primary readers because the former are assumed to have higher agreement on arbitration images, which are the most difficult to interpret. While a process with a single arbitrator may be necessary in studies with logistical constraints, this is not favored because variability among arbitrators means reproducibility between studies may be limited.

We found that a majority of CXRs at arbitration required consensus discussion to reach a final conclusion, which likely reflects the complexity of those CXRs. While initial blinded review by 2 arbitrators before a final discussion necessitates additional interpretations, feedback from our arbitrators suggests this may not be an increased workload compared to a discussion alone. Therefore, initial blinded review by 2 arbitrators followed by consensus discussion for discordant images appears to be an effective method to resolve the interpretation of CXRs that are discordant at the primary reading. Because we observed differences in the proportion of conclusions from consensus discussions that agreed with each arbitrators’ initial conclusions, future studies may benefit from ensuring these discussions are blinded to previous interpretations.

The PERCH study is the largest evaluation of the WHO methodology for the standardized interpretation of pediatric CXRs. Our results reinforce the reproducibility for detecting consolidation and the failure to achieve equally high concordance on other conclusions, including distinguishing normal from other infiltrates. The misclassification between these categories must be acknowledged in the analyses drawn from studies that use CXR findings. While limiting the number of final conclusion categories will improve observer agreement, the conclusion definition is the primary influence on agreement. Furthermore, resolving conclusions of discordant CXRs at primary reading should be done through additional independent arbitration readings, with any further discordance resolved through consensus discussion blinded to previous interpretations. Finally, the training process, quality control process, algorithm for drawing final conclusions, and the effect of prevalence on observer agreement all influence study results and need to be reported in detail so that any cross-study comparisons take these differences into consideration. Chest imaging continues to be an important element of pneumonia epidemiologic research, and efforts to improve image interpretation and observer variability, including use of computer-aided detection or other imaging techniques such as ultrasound, warrant additional evaluation.

## Supplementary Data

Supplementary materials are available at *Clinical Infectious Diseases* online. Consisting of data provided by the authors to benefit the reader, the posted materials are not copyedited and are the sole responsibility of the authors, so questions or comments should be addressed to the corresponding author.

## Supplementary Material

DAP63_Appendix_21NOV2016

## Notes

***Author contributions.*** N. F. developed and coordinated the chest radiograph interpretation process, performed analyses, interpreted results, and drafted the manuscript. M. D. K. and K. L. O. assisted with the analytic plan, interpretation of results, and drafting the manuscript. K. L. O., D. R. F., M. D. K., L. L. H., and S. A. M. conceived and designed the study and supervised study conduct. J. D. C. and M. D. C. designed and delivered the training and standardization process for the reading panel. B. B. K., M. D., B. E. E., R. I., A. K., F. B. M., N. M., D. P. M., M. M., K. N., C. O., M. S. O., J. S., and P. S. were members of the PERCH Chest Radiograph Primary Reading Panel. J. D. C., M. D. C., F. G., and V. M. were members of the PERCH Chest Radiograph Arbitration Panel. V. M. provided quality and safety assessments for radiology facilities at each study site. W. G. and C. P. were involved in study conduct and the coordination and management of the chest radiographs.

***Acknowledgments.*** We offer thanks to the Emmes Corporation, Rockville, Maryland, for the development of data management processes and online software used for the CXR interpretation process, and to Zhenke Wu for advice on biostatistics. We acknowledge the significant contributions of the PERCH Study Group and all PERCH investigators. We offer our gratitude to the members of the Pneumonia Methods Working Group and PERCH Expert Group for their time and lending expertise to assist the PERCH Study Group. See Supplementary Materials for a full list of names. We offer sincere thanks to the patients and families who participated in this study.

***Financial support.*** PERCH was supported by the Bill & Melinda Gates Foundation (grant number 48968 to the International Vaccine Access Center, Department of International Health, Johns Hopkins Bloomberg School of Public Health). N. F. was supported by an International Fulbright Science & Technology Fellowship from the U.S. Department of State.

***Supplement sponsorship.*** This article appears as part of the supplement “Pneumonia Etiology Research for Child Health (PERCH): Foundational Basis for the Primary Etiology Results,” sponsored by a grant from the Bill & Melinda Gates Foundation to the PERCH study of Johns Hopkins Bloomberg School of Public Health, Baltimore, Maryland.

***Potential conflicts of interest.*** M. D. K. has received funding for consultancies from Merck, Pfizer, and Novartis, and grant funding from Merck. L. L. H. has received grant funding from Pfizer and GlaxoSmithKline. S. A. M. has received honoraria for advisory board membership from the Bill & Melinda Gates Foundation, Pfizer, Medimmune, and Novartis; has received institutional grants from GSK, Novartis, Pfizer, Minervax, and the Bill & Melinda Gates Foundation; and has served on speaker’s bureaus for Sanofi Pasteur and GSK. K. L. O. has received grant funding from GSK and Pfizer and participates on technical advisory boards for Merck, Sanofi Pasteur, PATH, Affinivax, and ClearPath. All other authors report no potential conflicts. All authors have submitted the ICMJE Form for Disclosure of Potential Conflicts of Interest. Conflicts that the editors consider relevant to the content of the manuscript have been disclosed.
