# Supplementary material for: Standardized Interpretation of Chest Radiographs in Cases of Pediatric Pneumonia From the PERCH Study
Source: Clin Infect Dis. 2017 May 27;64(Suppl 3):S253–61. doi: 10.1093/cid/cix082 (PMC5447844; doi:10.1093/cid/cix082)
Supplement: DAP63_Appendix_21NOV2016 [file cix082_suppl_DAP63_Appendix_21NOV2016.pdf]

Supplemental Table 1: Overall observer agreement for primary readers across five conclusion categories. 'Reader one' and 'Reader two' represent random reader-pairs assigned to each CXR

		Reader Two					
		Only Consolidation	Other Infiltrate	Both	Normal	Uninter - pretable	**Total**
**Reader One**							
Only consolidation	N	294	119	149	79	67	**708**
	Row %	41.5	16.8	21.1	11.2	9.5	100
	Col %	41.6	10.6	29.2	5.6	15.3	17.0
Other infiltrate	N	79	361	90	296	48	**874**
	Row %	9.0	41.3	10.3	33.9	5.5	100
	Col %	11.2	32.3	17.6	21.1	11.0	21.0
Both	N	157	142	164	64	24	**551**
	Row %	28.5	25.8	29.8	11.6	4.4	100
	Col %	22.2	12.7	32.1	4.6	5.5	13.2
Normal	N	117	447	85	854	157	**1660**
	Row %	7.1	26.9	5.1	51.5	9.5	100
	Col %	16.6	40.0	16.6	61.0	35.9	39.8
Uninterpretable	N	59	49	23	107	141	**379**
	Row %	15.6	12.9	6.1	28.2	37.2	100
	Col %	8.4	4.4	4.5	7.6	32.3	9.1
**Total**	**N**	**706**	**1118**	**511**	**1400**	**437**	**4172**
	Row %	16.9	26.8	12.3	33.6	10.5	100
	Col %	100.0	100.0	100.0	100.0	100.0	100.0

Supplemental Table 2: Overall observer agreement for initial arbitration across five conclusion categories, excluding quality control CXRs.
'Arbitrator one' and 'Arbitrator two' represent random reader-pairs assigned to each CXR

		Arbitrator Two					
		Only Consolidation	Other Infiltrate	Both	Normal	Uninter - pretable	**Total**
**Arbitrator One**							
Only consolidation	N	121	46	108	25	40	**340**
	Row %	35.59	13.53	31.76	7.35	11.76	100
	Col %	41.87	7.42	32.63	3.25	11.49	14.42
Other infiltrate	N	62	276	93	140	71	**642**
	Row %	9.66	42.99	14.49	21.81	11.06	100
	Col %	21.45	44.52	28.1	18.18	20.4	27.23
Both	N	63	37	107	10	11	**228**
	Row %	27.63	16.23	46.93	4.39	4.82	100
	Col %	21.8	5.97	32.33	1.3	3.16	9.67
Normal	N	21	220	11	521	107	**880**
	Row %	2.39	25	1.25	59.2	12.16	100
	Col %	7.27	35.48	3.32	67.66	30.75	37.32
Uninterpretable	N	22	41	12	74	119	**268**
	Row %	8.21	15.3	4.48	27.61	44.4	100
	Col %	7.61	6.61	3.63	9.61	34.2	11.37
**Total**	**N**	**289**	**620**	**331**	**770**	**348**	**2358**
	Row %	12.26	26.29	14.04	32.65	14.76	100
	Col %	100	100	100	100	100	100

Supplemental Table 3. Observer agreement on individual conclusions (present or absent) for all 4172 images

	Observer agreement at primary readings and arbitration readings					
**Conclusion**	% Overall agreement	Kappa	(95% CI)	Bias Index[a]	Prevalence Index[b]	Adjusted Kappa[c]
Primary Readings (n=4172)						
Only Consolidation	80.2	0.30	(0.27 - 0.33)	0.0004	-0.66	0.60
Other Infiltrate	69.6	0.17	(0.14 - 0.20)	0.06	-0.52	0.39
Both	82.4	0.21	(0.18 - 0.24)	0.01	-0.75	0.65
Normal	67.6	0.31	(0.28 - 0.34)	0.06	-0.27	0.35
Uninterpretable	87.2	0.28	(0.24 - 0.31)	0.01	-0.80	0.74
Any Consolidation	77.3	0.46	(0.43 - 0.49)	0.01	-0.41	0.55
Arbitration Readings (n=2358)						
Only Consolidation	83.6	0.29	(0.25 - 0.33)	0.02	-0.73	0.67
Other Infiltrate	69.9	0.23	(0.19 - 0.27)	0.01	-0.46	0.40
Both	85.4	0.30	(0.26 - 0.34)	0.04	-0.76	0.71
Normal	74.2	0.43	(0.39 - 0.47)	0.05	-0.30	0.48
Uninterpretable	84.0	0.30	(0.26 - 0.34)	0.03	-0.74	0.68
Any Consolidation	83.5	0.56	(0.52 - 0.60)	0.02	-0.50	0.67

[a] Measures the difference in observers' proportions of positive and negative findings. Calculated as the difference in the proportion of positive interpretations for each observer and ranges from 0 if these proportions are equal to +1 if either observer provides all negative interpretations [1].
[b] Estimates the imbalance between overall positive and negative findings. Calculated as the difference in the probability of a positive interpretation and the probability of a negative interpretation and ranges from -1 when all interpretations are negative to +1 when all interpretations are positive [1].
[c] Adjusted for bias and prevalence [1].

Supplemental Table 4: Quality control images, comparing readers' initial concordant interpretations to conclusions from the arbitration process
(overall agreement 70.1%; Kappa 0.57)

	Final Arbitration Conclusion						
	Consolidation	Other infiltrate	Both	Normal	Uninterpretable	Total (col %) [a]	Disagreement (row %)
**Primary reading conclusion**							
Consolidation	20	1	5	0	3	29 (15.8%)	9 (31.0%)
Other infiltrate	2	17	2	13	1	35 (19.0%)	18 (51.4%)
Both	5	2	8	0	0	15 (8.2%)	7 (46.7%)
Normal	2	5	0	71	8	86 (46.7%)	15 (17.4%)
Uninterpretable	1	1	0	4	13	19 (10.3%)	6 (31.6%)
Total (row %)[a]	30 (16.3%)	26 (14.1%)	15 (8.2%)	88 (47.8%)	25 (13.6%)	184 (100%)	55 (29.9%)

[a] Pearson's χ^2, p=0.70 comparing distributions of conclusions from readers and arbitrators.

Supplemental Figure 1: Observer agreement across five chest radiograph conclusions for individual reader pairs (mean 49.7 CXRs interpreted per pair, range 41-60)

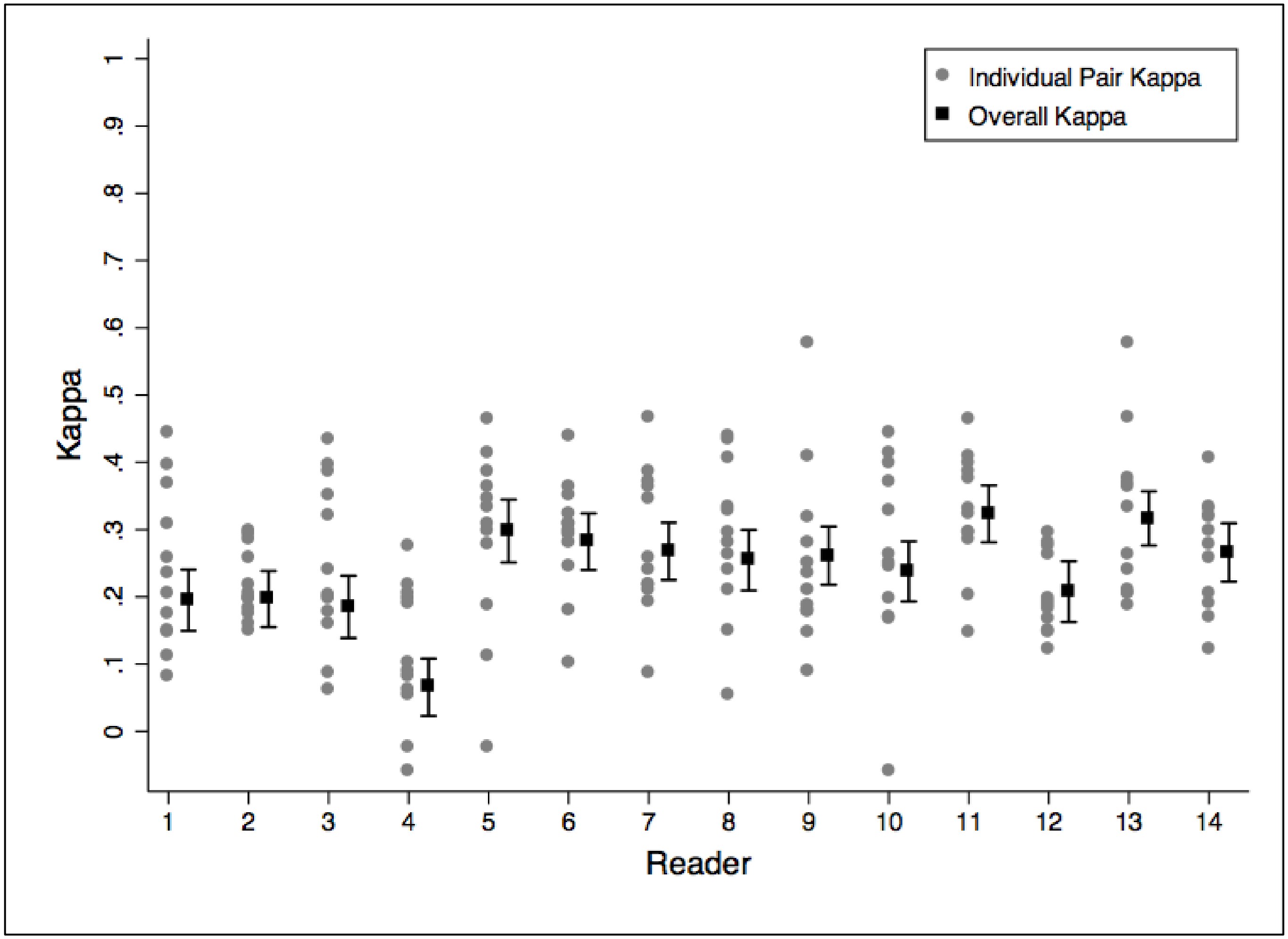

Supplemental Figure 2: Comparison of observer agreement for 'any consolidation' by site, excluding images for which either reader concluded as uninterpretable (n=3497, site range 198 – 782)

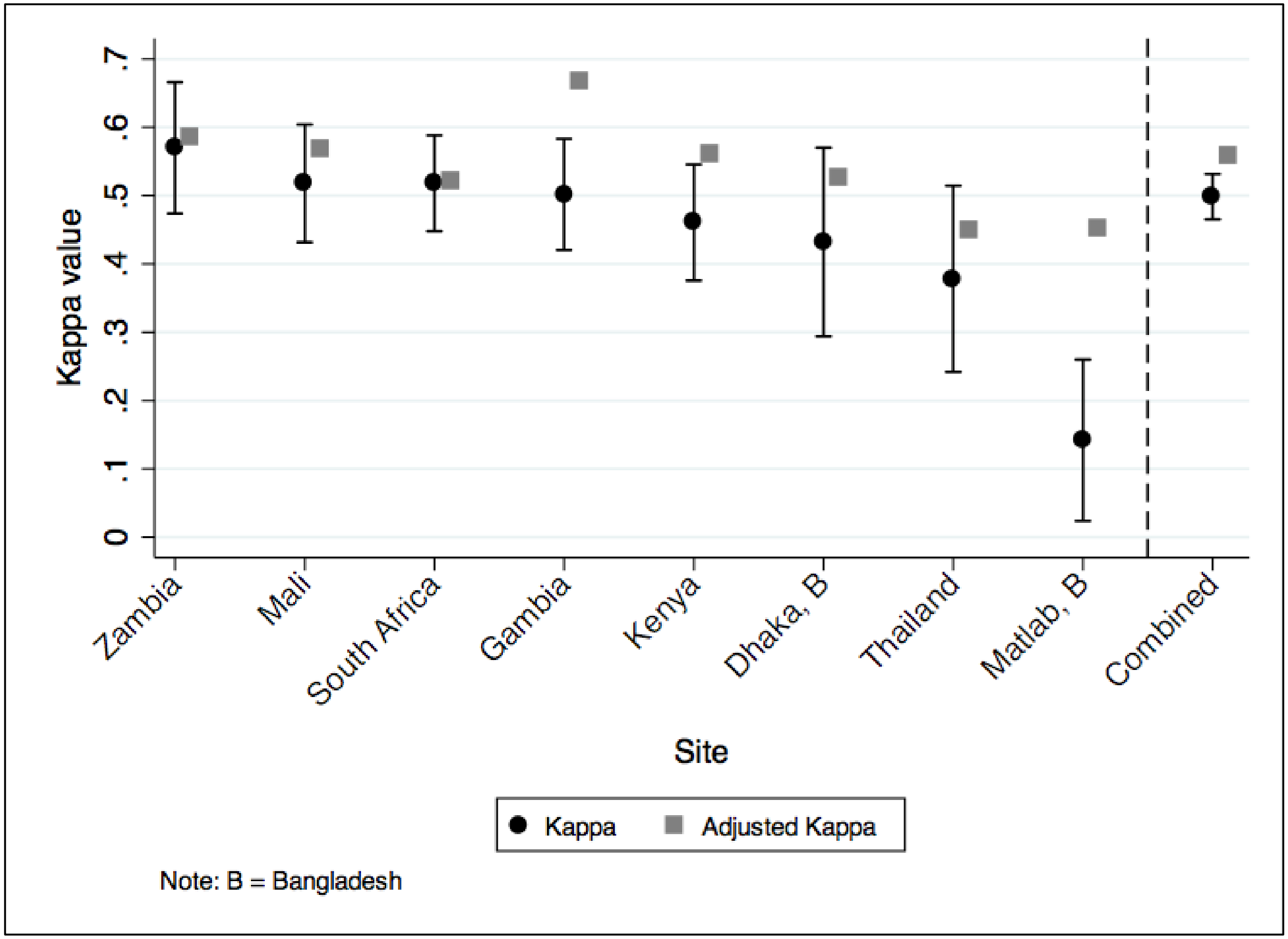

REFERENCES

1. Byrt T, Bishop J, Carlin JB. Bias, prevalence and kappa. J Clin Epidemiol **1993**; 46(5): 423-9.

The standardized interpretation of chest radiographs for PERCH
Appendix

***PERCH Study Group*.** **Johns Hopkins Bloomberg School of Public Health, Baltimore, Maryland**: Katherine L. O'Brien (PI), Orin S. Levine (Former PI, current affiliation Bill & Melinda Gates Foundation, Seattle, Washington**),** Maria Deloria Knoll (co-PI), Daniel R. Feikin (joint affiliation with Centers for Disease Control and Prevention, Atlanta, Georgia), Andrea N. DeLuca, Amanda J. Driscoll, Nicholas Fancourt, Wei Fu, Laura L. Hammitt, Melissa M. Higdon, E. Wangeci Kagucia, Ruth A. Karron, Mengying Li, Daniel E. Park, Christine Prosperi, Zhenke Wu, Scott L. Zeger; **The Emmes Corporation, Rockville, Maryland:** Nora L. Watson, **Nuffield Department of Clinical Medicine, University of Oxford, United Kingdom**: Jane Crawley; **University of Otago, Christchurch, New Zealand**: David R. Murdoch; **ICDDR, b, Dhaka and Matlab, Bangladesh**: W. Abdullah Brooks (site PI), Hubert P. Endtz, Khalequ Zaman, Doli Goswami, Lokman Hossain, Yasmin Jahan, Hasan Ashraf; **Medical Research Council, Basse, The Gambia**: Stephen R. C. Howie (site PI), Bernard E. Ebruke, Martin Antonio, Jessica McLellan, Eunice Machuka, Arifin Shamsul, Syed M.A. Zaman, Grant Mackenzie; **KEMRI-Wellcome Trust Research Programme, Kilifi, Kenya**: J. Anthony G. Scott (site PI and PERCH co-PI), Juliet O. Awori, Susan C. Morpeth, Alice Kamau, Sidi Kazungu, Micah Silaba Ominde; **Division of Infectious Disease and Tropical Pediatrics, Department of Pediatrics, Center for Vaccine Development, Institute of Global Health, University of Maryland School of Medicine, Baltimore, Maryland and Centre pour le Développement des Vaccins (CVD-Mali), Bamako, Mali**: Karen L. Kotloff (site PI), Milagritos D. Tapia, Samba O. Sow, Mamadou Sylla, Boubou Tamboura, Uma Onwuchekwa, Nana Kourouma, Aliou Toure; **Respiratory and Meningeal Pathogens Research Unit, University of the Witwatersrand , Johannesburg, South Africa**: Shabir A. Madhi (site PI), David P. Moore, Peter V. Adrian, Vicky L. Baillie, Locadiah Kuwanda, Azwifarwi Mudau, Michelle J. Groome, Nasreen Mahomed, **Thailand Ministry of Public Health – U.S. CDC Collaboration, Nonthaburi, Thailand**: Henry C. Baggett (site PI), Somsak Thamthitiwat, Susan A. Maloney (former site PI), Charatdao Bunthi, Julia Rhodes, Pongpun Sawatwong, Pasakorn Akarasewi (site co-PI, Ministry of Public Health); **Boston University School of Public Health, Boston, Massachusetts and University Teaching Hospital, Lusaka, Zambia**: Donald M. Thea (site PI), Lawrence Mwananyanda, James Chipeta, Phil Seidenberg, James Mwansa, Somwe wa Somwe, Geoffrey Kwenda.

PERCH Expert Group. William C. Blackwelder, Harry Campbell, John A. Crump, Adegoke Falade, Menno D de Jong, Claudio Lanata, Kim Mulholland, Shamim Qazi, Cynthia G. Whitney.

Pneumonia Methods Working Group. Robert E Black, Zulfiqar A Bhutta, Harry Campbell, Thomas Cherian, Derrick W Crook, Menno D de Jong, Scott F Dowell, Stephen M Graham, Keith P Klugman,

Claudio F Lanata, Shabir A Madhi, Paul Martin, James P Nataro, Franco M Piazza, Shamim A Qazi, and Heather J Zar.

PERCH Contributors:

Bangladesh: Kamrun Nahar, Arif Uddin Sikdir, Sharifa Yeasmin, Dilruba Ahmed, Muhammad Ziaur Rahman, Muhammad Yunus, Muhammad Al Fazl Khan, Muhammad Jubayer Chisti, Abu Sadat Muhammad Sayeem, Shahriar Bin Elahi, Mustafizur Rahman; **The Gambia:** Michel Dione, Emmanuel Olutunde, Peter Githua, Ogochukwu Ofordile, Rasheed Salaudeen, David Parker; **Kenya:** Shebe Mohamed, Siti Ndaa, Micah Silaba, Neema Muturi, Angela Karani, Sammy Nyongesa, Anne Bett, Daisy Mugo, Salim Mwarumba, Robert Musyimi, Andrew Brent, James Nokes, David Mulewa, Joyce Sande, John Odhiambo, Joshua Wambua, Nuru Kibirige, Caroline Mulunda, Hellen Mjalla, Norbert Katira, Karen Dama, Loice Masha, Christine Mutunga, Mwanajuma Ngama, Stephen Mangi, Riziki Anthony, Mwarua Yubu, Elijah Wakili, Benson Katana, Shoboi Mgunya, Emmanuel Mumba, Benedict Mver, George Kuria, Felix Githinji, Norbert Kihuha, Boniface Jibendi, Tahreni Bwanaali, Agustus Kea; **Mali:** Nana Kourouma, Aliou Toure, Mahamadou Diallo, Breana Barger-Kamate, Mariam Samake, Seydou Sissoko, Abdoul Aziz Maiga, Mariam Samake, Toumani Sidibe, Mariam Sylla, Aziz Diakite, Bassirou Diarra; **South Africa:** Azwidihwi Takalani, Andrea Hugo, Susan Nzenze, Ndulela Titi, Mmabatho Selela, Malebo Motiane, Minah Nkuna, Nonhlanhla Tsholetsane, Sibonsile Moya, Debra Katisi, Tondani Netshishivhe, Lerato Mapetla, Gudani Singo, Simphiwe Gasa, Cece Mgenge, Nozipho Mthunzi, Nombulelo Monedi, Tanja Adams, Shafeeka Mangera, Jeannette Wadula, Peter Tsaagane, Jenifer L. Vaughan, Sakina Loonat, Martin Hale, Sugeshnee Pather, Mariëtte Middel, Siobhan Trenor, Palesa Morailane, Ntombi Maya, Rene Sterley, Charné Combrinck, Given Malete, Lerato Qoza, Grizelda Liebenberg, Hendrik van Jaarsveld, Zunaid Kraft, Lisa-Marie Mollentze, Lourens Combrinck, Tsholofelo Mosome; **Thailand:** Sununta Henchaichon, Dr. Tussanee Amornintapichet, Dr. Somchai Chuananont, Toni Whistler, Juraiporn Ratanodom, Patranuch Sapchookul, Ornuma Sangwichian, Sirirat Makprasert, Manoon Hirunsalee, Possawat Jorakate, Anek Kaewpan, Duangkamol Siludjai, Apiwat Lapamnouysup, Dr. Wantana Paveenkittiporn, Waraporn Ubonphen, Dr. Peera Areerat, Ms.Yupapan Wannachaiwong, Ms Tewa Faipet, Ms Punnat Natnarakorn, Ms Ahchanan Sacharone, Mr.Winai Makmool, Ms. Kanlaya Sornwong, Ms. Promporn Sansuriwong, Ms. Ratchanida Potiya, Ms. Wasana Hongsawong, Ms.Wipa Matchaikhen, Ms. Thatsanawan Chaiyabil, Ms.Piyapai Wannarach, Ms Chamaiporn Wadeesirisak, Mr. Yuttapong Norapet, Mattana Bangkung, Mr. Barameht Piralam, Sathapana Naorat, Anchalee Jatapai, Prasong Srisaengchai, Dr. Leonard Peruski, Ms.Dawan Phaensoongnoen, Ms.Tussaaorn Klangprapan, Ms.Narawadee Dumrongdee, Ms.Atchara Srithongkham, Mr. Piyawut Noinont, Ms. Pornthip Kamlee, Ms.Siyapa Mongkornsuk; **Zambia:** Justin Mulindwa, Musaku Mwenechanya, John Mwaba, Magdalene

Mwale, Julie Duncan, Kazungu Siazele, Muntanga Mapeni, Emily Hammond; **Canterbury Health Laboratory, Christchurch, New Zealand:** Rose Watt, Shalika Jayawardena.
